# Microbial community structure of soils in Bamenwan mangrove wetland

**DOI:** 10.1038/s41598-019-44788-x

**Published:** 2019-06-10

**Authors:** Min Liu, Huiqin Huang, Shixiang Bao, Yuhe Tong

**Affiliations:** 1 Hainan Tropical Ocean University, Sanya, China; 2 Institute of Tropical Biosciences and Biotechnology, Chinese Academy of Tropical Agricultural Sciences, Haikou, China

**Keywords:** Soil microbiology, Biodiversity

## Abstract

Microbial community diversity and composition are important for the maintenance of mangrove ecosystem. Bacterial and archaeal community composition of the Bamenwan Mangrove Wetland soil in Hainan, China, was determined using pyrosequencing technique. Bacterial community composition presented differences among the five soil samples. Rhizobiales with higher abundance were observed in inner mangrove forest samples, while Desulfobacterales were in the seaward edge samples, and Frankiales, Gaiellales and Rhodospirillales in the landedge sample. For archaea, Crenarchaeota and Euryarchaeota dominated in five samples, but the proportion in each samples were different. Dominant archaeal community composition at the order level was similar in the seaward edge samples. The dominant archaeal clusters in the two inner mangrove forest samples were different, with Soil Crenarchaeotic Group (SCG) and Halobacteriales in sample inside of *Bruguiera sexangula* forest and SCG, Methanosarcinales and Marine Benthic Group B (MBGB) in sample inside of *Xylocarpus mekongensis* forest. The dominant archaeal clusters in land sample were unique, with Terrestrial Group and South African Gold Mine Group 1. The metabolic pathways including metabolism, genetic information processing, environmental information processing, cellular processes, organismal systems and human diseases were all detected for bacterial and archaeal functional profiles, but metabolic potentials among five samples were different.

## Introduction

Mangroves are intertidal estuarine wetlands ecosystem along the tropical and subtropical coasts, covering approximately 60 to 75% of the global coastline^1^. Mangrove ecosystems serve a variety of important ecological and economic functions, including protecting coastlines from storm damage and erosion, degrading environmental contaminants, and providing nursery habitats for numerous aquatic organisms^2–6^. Despite the known ecological importance of mangrove forests, human activities place the forests under the rising threat of extinction^3^.

Mangrove ecosystems are one of the most productive ecosystems in the world. They are characterized by high levels of salinity, high redox potential and organic matter contents, and high rates of nutrient recycling^1,7,8^. Under such unique environmental conditions, the mangrove habitat contains abundant and characteristic microbial resources^1,7,8^, which make mangrove as the hotspots for microbial diversity. The mangrove microbiota is composed of a combination of terrestrial soil, marine and freshwater microorganisms^1,7,8^. These microorganisms play critical roles in mangrove ecosystem maintenance and function^1,7,8^. Microbes contribute to biogeochemical cycles and serve as a primary nutrition source to plants and animals^8^. Microbial diversity and activity are essential for the productivity, conservation, and recovery of mangroves^9^. Microbial diversity in mangrove soil is influenced by biogeographical, ecological, and anthropogenic factors^1,8^. Additionally, mangrove plants and microorganisms have a close relationship. For example, many microorganisms are beneficial to the growth of mangrove plants. Nitrogen-fixing bacteria, phosphate-solubilizing bacteria, and sulfate-reducing bacteria have been isolated and cultured from mangrove soils^10,11^. Mangrove plants provide microbial colonization sites and nutrients for microbial growth^12,13^. Despite these known connections, the details of rhizosphere effect of mangrove plants on the microbial communities remain unclear due to limited studies on these microenvironments.

Many microbiological studies in mangrove ecosystems have been reported in the last few years. Some of these studies have isolated and identified microbial strains with the potential for biotechnical use^14^. Some studies have characterized the microbial groups present in the mangrove ecosystems, such as archaea^14–16^, fungi^17^ and cyanobacterial^18^, as well as characterized the diversity of special microbial populations involving nutrient biogeochemical cycling, such as nitrogen^19^, sulfur^20^ and carbon^21^. Some studies have characterized bacterial community structures in rhizosphere and bulk mangrove soils^22,23^. Previous studies have shown that diverse and variable microbial communities harbored in mangrove ecosystem^24^. The anaerobic and high salinity environment of the mangrove wetlands provide conditions for archaea to thrive, so the domain archaea is particularly important for mangrove ecosystem^25,26^. However, previous studies have mainly focus on bacterial communities, so there was a lack of information regarding archaeal communities in mangrove soils. The archaeal communities in mangrove soils have been examined using clone libraries^19,20,27–32^, but details of archaeal community diversity in mangrove wetlands could not be revealed using this method. This is the first characterization of the archaeal community within mangrove ecosystem soil that has been reported using data from high-throughput next generation sequencing. Next generation sequencing, like pyrosequencing and Illumina sequencing provides a relatively detailed picture of microbial communities compared to other methods. In this study, we describe details of bacterial and archaeal communities that exist within different sites of the mangrove wetland using pyrosequencing. These results may be helpful for guidance to isolate bacteria of interest within these distinct sites.

## Results

### Sequencing and quality control

A total of 94013 and 100585 valid reads, for bacteria and archaea, respectively, were obtained for five soil samples in the Bamenwan Mangrove Wetland (Table 1). The average read lengths for bacterial and archaeal samples were 597 bp and 499 bp, respectively. Table 1 shows that 77–82% of the raw reads met quality and length standards for bacteria. After initial quality check mentioned above, the chimera, Achaea and singleton reads were also checked and filtered out. Finally, 7951, 8072, 7757, 8992 and 8039 effective bacterial sequences were extracted from each of the five samples, respectively, for use in downstream bioinformatic analyses (Table 1). For archaea, 86–89% of the raw reads met quality and length standards. 13640, 3056, 15636, 16203 and 12819 effective archaeal sequences were obtained from each of five samples, respectively, for use in downstream bioinformatic analyses after the initial quality check (Table 1).

**Table 1:** Sequences from the five samples and diversity indices in this study.

Sample name	Valid reads	Trimed reads	Effective sequences	OTUs	Unique	Richness	ACE	Shannon Index (H’)	Coverage (%)
				Total		Chao 1			
**Bacteria**									
BM1	15991	13067	7951	668	85	786	777	5.74	98.2
BM2	18420	14816	8072	715	121	751	756	5.80	98.9
BM3	18793	14763	7757	775	72	890	880	5.73	97.9
BM4	20188	15753	8992	870	91	968	969	5.85	98.2
BM5	20621	15803	8039	427	169	461	454	5.10	99.4
**Archaea**									
BM1	20201	17700	13640	634	255	653	656	5.35	99.6
BM2	20121	17845	13056	645	243	663	671	5.15	99.5
BM3	19529	17313	15636	379	89	414	415	2.92	99.6
BM4	21937	19413	16203	484	123	517	525	3.94	99.5
BM5	18797	16077	12819	123	74	125	126	3.34	99.9

### Bacterial diversity indices and community structure

**Figure 1:**
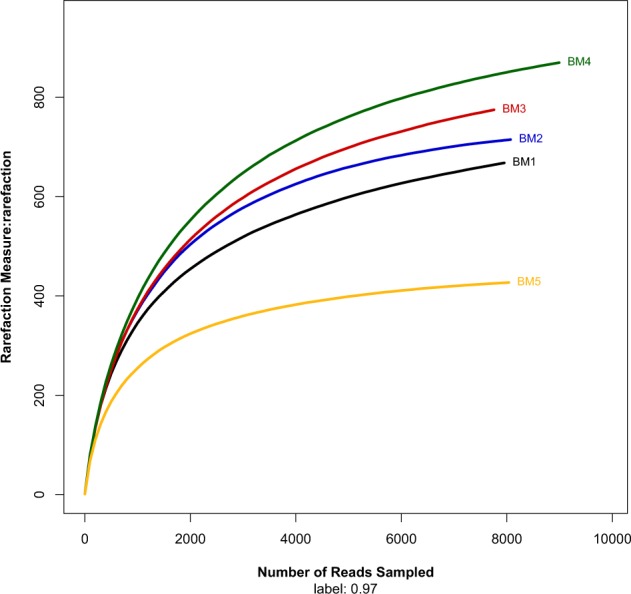
Rarefaction curves for OTU for bacteria of the five sediment samples in the mangrove ecosystem at cutoff level of 3% created by using Mothur (version v.1.30.1).

The effective sequences were clustered into operational taxonomic units (OTUs) using 97% similarity cutoff with UPARSE, which is a method with a focus on reducing OTU inflation. The OTU number range for all five samples was 427 to 870 OTUs using a distance cutoff level of 3% (Table 1). The BM5 sample contained the lowest OUT number. Diversity was highest in the BM4 sample and the lowest in BM5 sample (Table 1, Fig. 1). The Shannon diversity index also revealed that the BM5 sample had the lowest bacterial diversity among the five samples (Table 1). The rarefaction curves of the five samples did not reach a plateau, indicating that the data did not contain enough sequence depth to ascertain the full bacterial diversity (Fig. 1).

**Figure 2:**
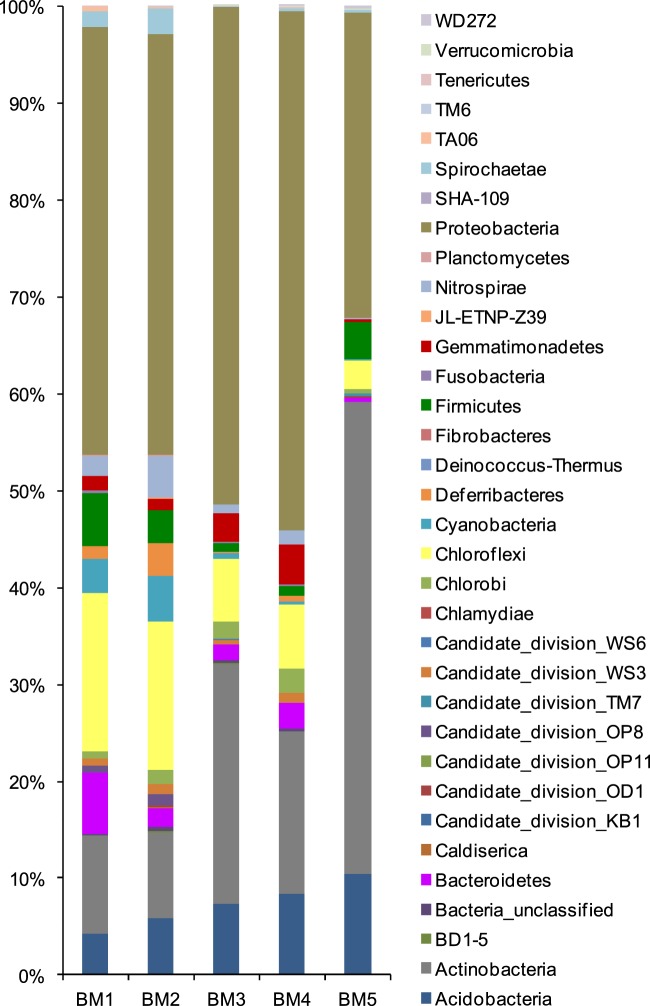
Relative abundance of bacterial groups at the phylum level in the five sediment samples in mangrove ecosystem.

Thirty-four phyla were detected within the five samples. The top 10 phyla present in the samples were Proteobacteria, Actinobacteria, Chloroflexi, Acidobacteria, Firmicutes, Bacteroidetes, Gemmatimonadetes, Cyanobacteria, Nitrospirae, and Chlorobi (Fig. 2). Proteobacteria was the predominant phylum in all five samples. The highest percentages of Proteobacteria were detected in the samples BM3 and BM4 (51.40% and 53.53% of the total sequences in each sample, respectively). The lowest percentage of Proteobacteria was detected in sample BM5 (31.53%) (Table S1). Actinobacteria was the second most abundant phylum, with the highest percentage of 48.40% in the sample BM5 and lower percentages of 10.10% and 8.86% in the samples BM1 and BM2, respectively (Table S1). The percentages of Chloroflexi were higher in the samples BM1 and BM2, (16.30% and 15.28%, respectively), than those of other three samples, (which ranged from 2.99–6.54%) (Table S1). Acidobacteria were detected in greater abundance in the sample BM5 (10.37%) than in the other four samples (4.25–8.43%) (Table S1). The percentages of Cyanobacteria and Deferribacteres in the samples BM1 (3. 55% and 1.31%, respectively) and BM2 (4.78% and 3.39%, respectively) were higher than those of the other three samples (0.53% and 0.23% on average, respectively) (Table S1). Gemmatimonadetes were detected in greater numbers in the samples BM3 and BM4 (2.98% and 4.07%, respectively) than those of the other three samples (which ranged from 0.27–1.50%) (Table S1). In summary, the percentage abundance of different bacterial phyla differed between the five samples. Compared with the other four samples, the composition of the bacterial community in sample BM5 was distinct, and dominated by Actinobacteria (representing 48.70% of the total sequences), Proteobacteria (31.53%), and Acidobacteria (10.37%). However, some predominant bacterial clusters were similar between some samples. For example, the predominant phyla in samples BM1 and BM2 were similar. These samples were composed of Proteobacteria (with 44.20% and 43.27% of the total sequences in each sample, respectively)), Chloroflexi (with 16.30% and 15.28%, respectively) and Actinobacteria (with 10.10% and 8.86%, respectively). The bacterial community composition in samples BM3 and BM4 were both mainly dominated by Proteobacteria (with 51.40% and 53.53% of the total sequences in each sample, respectively), Actinobacteria (with 24.82% and 16.70%, respectively), Acidobacteria (with 7.30% and 8.43%, respectively) and Chloroflexi (with 6.54% and 6.57%, respectively) (Table S1).

In the Proteobacteira phylum, Alphaproteobacteria (40.51% of the Proteobacteira phylum on average), Deltaproteobacteria (32.89%) and Gammaproteobacteria (23.01%) were the main three classes detected in the five samples (Table S2). However, the proportion of these three classes differed among the five samples. Alphaproteobacteria was present in the highest abundance (77.99% in the Proteobacteira phylum) in sample BM5, and in the lowest abundance (11.34%) in sample BM2 (Table S2). Deltaproteobacteria was detected in the highest abundance (62.67%) in sample BM2, and in the lowest abundance (3.27%) in sample BM5 (Table S2). Gammaproteobacteria was detected in the highest abundance (36.25%) in sample BM1, and in the lowest abundance (16.61%) in sample BM5 (Table S2).

Within Alphaproteobacteria, the dominant orders were Rhizobiales and Rhodospirillales, with the highest abundance detected in samples BM3 (21.62% of total sequences) and BM5 (14.96% of total sequences), respectively (Table S3). The majority of Deltaproteobacteria sequences belonged to Desulfobacterales and Syntrophobacterales, which were both present in high abundance in samples BM1 and BM3 (accounting for 13.80% and 3.83%, respectively) compared to the other three samples (accounting for 3.15% and 0.73%, respectively) (Table S3). Within Gammaproteobacteria, the dominant orders were Xanthomonadales and Chromatiales. Xanthomonadales were detected in all five samples with percent abundances ranging from 1.92–7.23%. Chromatiales were not detected in sample BM5 but presented in the other four samples at similar percentages ranging from 2.66% to 3.46% (Table S3). Acidimicrobiales, Frankiales, Gaiellales, Solirubrobacterales, as the major orders of Actinobacteria, were present in high proportions in sample BM5 (especially for Frankiales, with a percent abundance of 22.20%) (Table S3). In summary, In summary, the bacterial community composition differed between the five samples the level of order. The main orders detected in sample BM1 were Desulfobacterales (10.51%), Xanthomonadales (7.23%), Anaerolineales (5.94%), Rhizobiales (5.82%) and Acidimicrobiales (4.50%). The main orders detected in sample BM2 were Desulfobacterales (17.08%), Anaerolineales (7.04%), Nitrospira (4.35%), Rhizobiales (3.90%) and Syntrophobacterales (3.79%). The main orders detected in sample BM3 were Rhizobiales (21.62%), Acidimicrobiales (7.77%), Solirubrobacterales (7.14%) and Gaiellales (6.41%), Xanthomonadales (4.55%). The main orders detected in sample BM4 were Rhizobiales (19.54%), Desulfobacterales (6.29%), Gaiellales (5.94%), Acidimicrobiales (4.10%) and Solirubrobacterales (4.06%). The main orders detected in sample BM5 were Frankiales (22.20%), Gaiellales (15.31%), Rhizobiales (8.66%), Rhodospirillales (14.96%) and Solirubrobacterales (5.25%) (Table S3).

The results of principal component analysis (PCA) at OTUs levels indicated that the five samples could be separated into three groups (Fig. S1a). The BM1 and BM2 samples were grouped together, and the BM3 and BM4 samples clustered together. Sample BM5 was separated from the other four samples. The results of PCoA analysis using the weighted and unweighted UniFrac metrics gave results that were similar to the PCA results (Fig. S1b,c).

### Archaeal diversity indices and community structure

**Figure 3:**
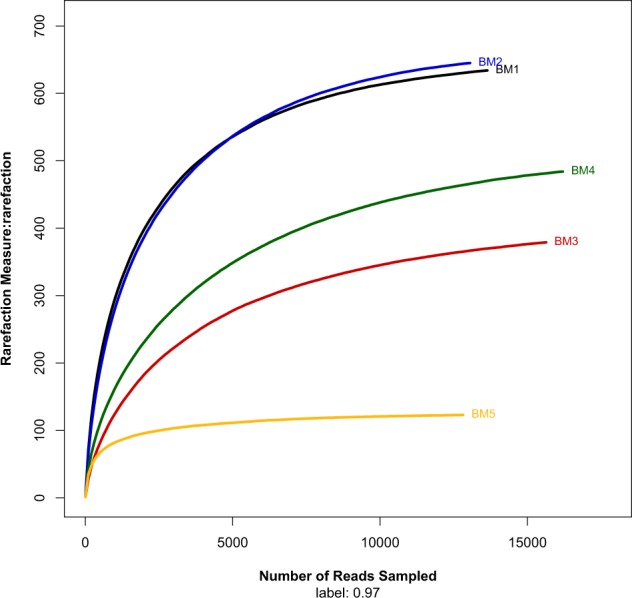
Rarefaction curves for OTU for archaea of the five sediment samples in the mangrove ecosystem at cutoff level of 3% created by using Mothur (version v.1.30.1).

For archaea, the OTU number, the taxon richness level reflected by Chao1 and ACE estimators, and the Shannon diversity index were relatively higher in samples BM1 and BM2, and lowest in sample BM5 (Table 1). The archaeal richness and diversity were higher in the soil inside *Xylocarpus mekongensis* mangrove forest (sample BM4) than inside the *Bruguiera sexangula* mangrove forest (sample BM5) (Fig. 3). Like the bacterial rarefaction curves, the archaeal rarefaction curves of the five samples did not reach a plateau, indicating the sequencing depth of these samples was not sufficient to fully assess archaeal diversity (Fig. 3).

**Figure 4:**
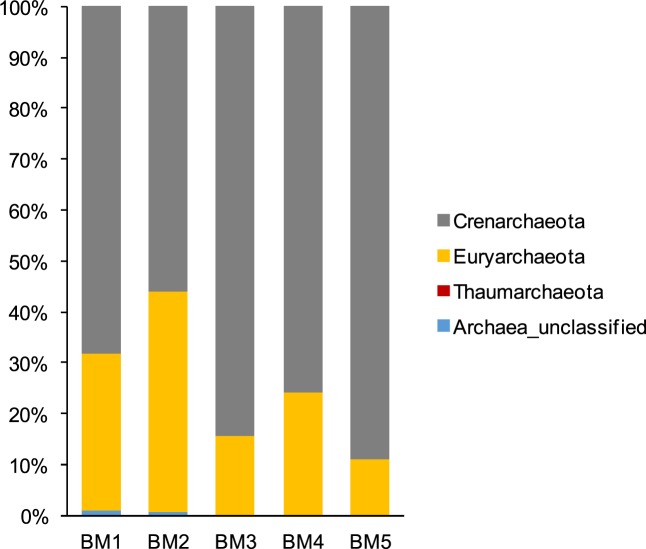
Relative abundance of archaeal groups at the phylum level in the five sediment samples in mangrove ecosystem.

Three different archaeal phyla, Crenarchaeota, Euryarchaeota and Thaumarchaeota, were detected in this study (Fig. 4). Crenarchaeota and Euryarchaeota were dominat in the five samples, but the proportion of each phylum differed between samples. The relatively higher proportion of Euryarchaeota were in samples BM2 (43.25%) and BM1 (30.85%), and then in samples BM4 (24.06%) and BM3 (15.66%), and the lowest proportion in BM5 (11.11%) (Fig. 4). The observed trend in Crenarchaeota abundance was the reverse of the trend observed in Euryarchaeota. The highest abundance was detected in sample BM5 (88.86%), then in samples BM3 (84.34%) and BM4 (75.76%), and relatively lower proportion in the samples BM1 (68.06%) and BM2 (55.97%) (Fig. 4). Crenarchaeota were only observed at low abundance in sample BM1 (0.02%). The Halobacteriales, Methanosarcinales and Thermoplasmatales orders dominated within Euryarchaeota, with the highest abundances observed in BM2 (20.8%), BM4 (13.9%) and BM2 (18.4%), respectively (Table S4). The observed Crenarchaeota mainly belonged to the Marine Benthic Group B (MBGB), Miscellaneous Crenarchaeotic Group (MCG), Soil Crenarchaeotic Group (SCG), South African Gold Mine Group 1(SAGMCG-1), Group C3, and the Terrestrial Group (Table S4). MCG was found to be relatively abundant in BM1 (36.20%) and BM2 (16.80%), and followed in BM3 (2.40%) and BM4 (8.40%). SCG was detected at high abundance in BM3 (75.60%) and BM4 (43.00%), and at low abundance in the other three samples (ranging from 0.90% to 2.40%) (Table S4). MBGB and Group C3 accounted for a large proportion of samples BM1, BM2 and BM4, but were absent in the sample BM5. Terrestrial Group and SAGMCG-1, as the predominant orders with 68.70% and 14.80%, respectively, were only detected in the sample BM5 (Table S4).

In summary, similar patterns in abundance in archaeal community composition at the order level were detected in samples BM1 and BM2. Both samples contained MCG, MBGB, Halobacteriales, Thermoplasmatales and Group C3. In these two samples, however, the percentages of MCG and Halobacteriales showed the reverse trend (Table S3). The most abundant archaeal clusters differed between the two mangrove forest samples, with SCG (75.60%) and Halobacteriales (8.10%) present in BM3 and SCG (43.00%), Methanosarcinales (13.90%) and MBGB (12.60%) present in BM4. Compared to the former four samples, the dominant archaeal clusters in BM5 were unique, containing Terrestrial Group (68.70%) and SAGMCG-1 (14.80%) (Table S4).

The results of PCA at OTUs levels indicated that the five samples could be separated into four groups (Fig. S1d). BM5 was separated from the other four samples. The BM1 and BM2 samples were grouped together, and the BM3 and BM4 samples clustered with one another. Similar results were also found in PCoA analysis using the weighted and unweighted UniFrac metrics (Fig. S1e,f). The PCA and PCoA results were consistent with the community composition results mentioned above.

### Functional properties predicted by PICRUSt

**Figure 5:**
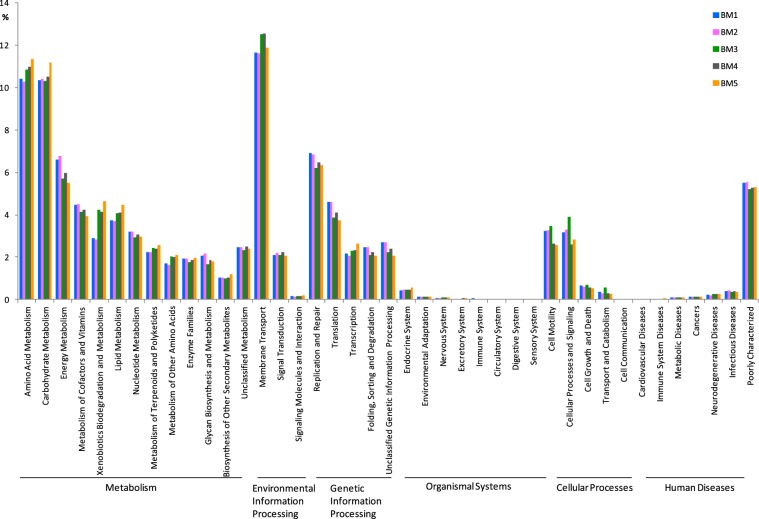
PICRUSt analysis of predicted metagenomes generated by using the bacterial 16 S rDNA data of the five sediment samples in mangrove ecosystem.

**Figure 6:**
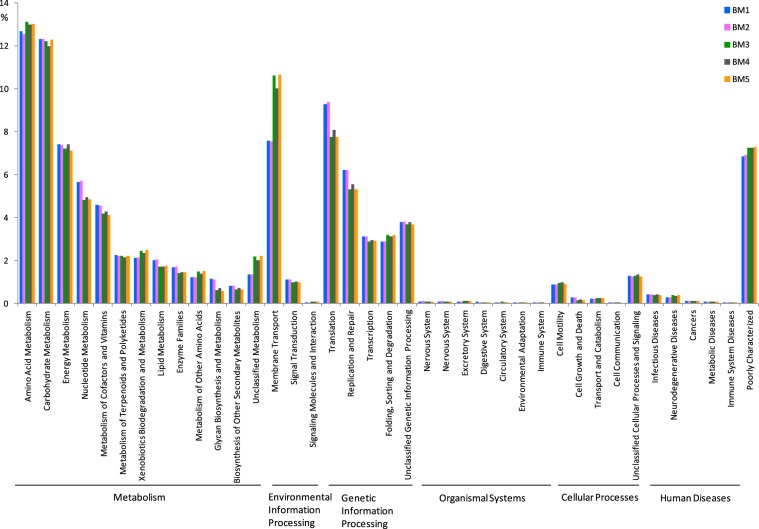
PICRUSt analysis of predicted metagenomes generated by using the archaeal 16S rDNA data of the five sediment samples in mangrove ecosystem.

PICRUSt was used to explore the different metabolic potentials among the samples from different sites in the mangrove wetland. The metabolic pathways including metabolism, genetic information processing, environmental information processing, cellular processes, organismal systems and human diseases were all detected for bacterial and archaeal functional profiles (Figs 5 and 6). For bacteria, PICRUSt analysis revealed that metabolic pathways such as energy metabolism, glycan biosynthesis and metabolism, replication and repair and translation were more abundant in the seaward edge samples, and membrane transport pathway was more abundant in inner samples, and pathways related to amino acid metabolism, carbohydrate metabolism, xenobiotics biodegradation and metabolism, lipid metabolism and transcription were more abundant in the landedge sample (Fig. 5). For archaea, the pathways such as nucleotide metabolism, metabolism of cofactors and vitamins, lipid metabolism, glycan biosynthesis and metabolism, translation and replication and repair were more abundant in seaward edge samples, and amino acid metabolism, membrane transport, and xenobiotics biodegradation and metabolism were more abundant in the other three samples (Fig. 6).

## Discussion

Soil microbial communities are active and vital components of mangrove ecosystems and play essential roles in nutrient biogeochemical cycling, organic matter remineralization and contaminant degradation^33^. The microbial communities in the mangrove soil are significantly affected by bio-geographical, anthropological, and ecological factors. These factors include the food web within the mangrove ecosystem, nutrient cycling, and the presence of inorganic or organic compounds in the soil^34^. Recently, microbial diversity and community structures have been studied in several mangrove ecosystems, such as those located in Brazil^35^ and Hong Kong^36^. In this study, a barcoded pyrosequencing analysis of 16S rRNA gene was performed to characterize the bacterial and archaeal communities present within five different sites in Bamenwan Mangrove Wetland in Hainan, China.

Metagenomic data collected from mangrove soil samples in Sao Paulo State, Brazil revealed an abundance of Proteobacteria (47.1–56.3%), Firmicutes (10.5–13.8%), Actinobacteria (5.4–12.2%), and Bacteroidetes (3.8–11.8%)^30^. In this study, Proteobacteria (31.53–53.53%), Actinobacteria (8.86–48.70%), Acidobacteria (4.25–10.37%), and Chloroflexi (2.99–16.30%) were dominant bacterial groups detected in the two samples taken from inside of the mangrove forest. In the previous two studies, Proteobacteria and Actinobacteria were both the predominant bacterial groups, which might suggest these bacterial groups are cosmopolitan inside of mangrove environments. However, differences between the abundant bacterial groups detected in these studies were also obvious. For example, Firmicutes and Bacteroidetes were detected at 0.76–5.43% and 0.40–6.28%, respectively, in the present study. These differences might be due to biogeographical, ecological (such as different mangrove plant community composition), and anthropogenic factors (such as nearby aquiculture and urbanization).

In this study, bacterial community structure among soil samples from the seaward edge, the inner and the landedge of the Bamenwan mangrove wetland presented differences. The two previous studies^26,31^ reported that spatial differences had a significant effect on bacterial community composition. For example, Actinobacteria, Acidobacteria, Nitrospirae, and Verrucomicrobia were enriched in the nutrient-rich inner mangrove soils, but Proteobacteria and Deferribacterias were enriched in the outer mangrove soils. Jiang *et al*.^35^ compared the bacterial communities in the mangrove wetland with that of freshwater reservoirs and marine sediments, and found that diverse groups of bacteria with functions related to the primary production were enriched in the mangrove wetland. These studies suggest that the intertidal mangrove wetland has a unique bacterial community. The mangrove wetland may be unique at the level of bacterial composition because it is the only forest ecosystem in marine environments, making it a unique intertidal ecosystem. The mangrove plant root exudates secreted from the roots of plants into the rhizosphere and surrounding sediment may act as nutrient sources or inhibitors for microbial populations^31^.

Rhizobiales was observed in the rhizospheres of mangrove trees^26^ and bulk soils around the mangrove tree roots. This suggests Rhizobiales may be common in mangrove soil bacterial communities and the presence of Rhizobiales is not restricted to one mangrove tree species. Rhizobiales are comprised of nitrogen-fixing bacteria and plant symbionts. Recently, a genomic analysis indicated that Rhizobiales are very metabolically versatile and are capable of degrading aromatic compounds^26^. Our results indicate the proportion of Rhizobiales in the two inner mangrove wetland samples is much higher than the proportion in the seaward and landedge samples. This suggests that Rhizobiales contribute to nitrogen-fixation in mangrove soils and promote mangrove plant growth. In addition, some specific orders are present within the two samples, such as Anaerolineales, Gemmatimonadales, Nitrospira, Syntrophobacterales detected in the soil sample from *Xylocarpus mekongensis* forest. This suggests mangrove plants have some impact on bacterial community composition not only in the rhizosphere, but also in the bulk soils around plant roots. Previous studies have illustrated that soil physiochemical properties, such as salinity, soil pH, nutrient concentration and composition, and root exudates, are the major factors influencing the activity and microbial community of mangrove soils^31,37^.

Mangrove soils are anoxic environments^26^, with high levels of salinity, high redox potential, and high amounts of organic matter and sulphates^38^. Accordingly, sulphur transformation is one of most active chemical cycles in the mangrove ecosystem^39–42^. Sulphur transformation, in its various forms, is mediated by microorganisms^1,43^. Microbial sulfate reduction is important for anaerobic degradation of organic matter in marine soils^7,44^ and is often mediated by bacterial clusters belonging to Deltaproteobacteria, specifically, Desulfobacterales^44,45^. In this study, Desulfobacterales were the dominant cluster in the four soil samples (including the seaward edge and the inner) except the landedge sample (BM5). This result was consistent with previous results as described abundant in mangrove soils^46^. These results suggested that Desulfobacterales as dominant clusters contributed greatly to sulfur transformation in the mangrove ecosystem. The results additionally suggest that sulfate reduction may be a primary pathway for anaerobic degradation of organic matter in mangrove soils. Desulfobacterales was also found to be abundant in polluted sites^47^, where they are associated with anaerobic degradation of hydrocarbons^48,49^. Taketani *et al*.^50,51^ report that Deltaproteobacteria are stimulated by oil pollution. These results suggest that mangrove and adjacent coastal ecosystems not only contain high levels of organic matter, but also high levels of pollutants. Indeed, it has been reported that the offshore areas (including mangrove ecosystems) of China are heavily polluted with organic contaminants and heavy metals due to the rapid economic development of coastal regions^52^. In addition, some other known sulfate-reducing bacterial clusters were also detected in this study, such as Syntrophobacterales presented in the seaward edge and the inner of mangrove forest soils, which indicated the diversity of sulphidogenic prokaryotes in mangrove ecosystem^53^.

In this study, bacterial clusters involved in other types of biogeochemical cycling, like phosphate and nitrogen cycling, were also observed. Nitrospira detedcted in all samples in this study, was described as nitrite-oxidizing bacteria in nitrogen cycle^51^. Rhizobiales as nitrogen-fixing bacteria were also detected in all samples. Deferribacteraleswere detected in the mudflat, marine edge and mangrove forest soils, and were considered as potential heterotrophic nitrate reducers^50,51^.

Frankiales, belonging to Actinobacteria, was the most dominant order detected, with up to 22.20% in the landedge sample (BM5). *Acidothermus* was the major genus of Frankiales (Table S3). This genus contains a single species, namely *A. cellulolyticus*, which is thermophilic, acidophilic and could produce many thermostable cellulose-degrading enzymes^54^. Gaiellales, a member of order Actinobacteria, was also abundant in the landedge sample (BM5). However, little is known about the physiology of Gaiellales. It is a novel order within the class Actinobacteria^55^. Rhodospirillales, the third most abundant order detected in the landedge sample, is comprised of many acetic acid-producing bacteria. These bacteria may be responsible for the low pH of the land sample soil.

Many bacterial members from Bacillales are considered beneficial to plant growth and also have protective effects against diseases^56^. Bacillales account for 1.35% of the bacteria detected in the present study, on average. Some isolates from the root and rhizosphere soil in the mangrove environment were phosphorus-solubilizing bacteria^57,58^. Bacillales may play a special role in mangrove ecosystems by engaging in long term promotion of plant growth. It is possible the Bacillales promote growth by producing endospores under stressful environmental conditions and by secreting large quantities of enzymes, such as phytase, a critical component of the phosphorous cycle^59^.

As showun from the previous studies, MBGB and MCG are ubiquitous in marine environments^60–62^. In the present study, a higher relative abundance of the two archaeal clusters was observed in seaward edge samples. No sequences were observed in the landedge sample. This distribution pattern was consistent with the previous reports. It is hypothesized that MCG and MBGB are to be anaerobic heterotrophs that consume buried carbon^63–65^. Lloyd *et al*.^66^ reports that MCG plays an important role in degradation of detrital proteins in anoxic marine soils. Based on these studies, the prevalence of MBGB and MCG in coastal sediments could contribute to the degradation of organic matter.

## Methods

### Site characterization and sample collection

Surface soil (top 30 cm) samples were collected from the Bamenwan mangrove wetland in Hainan, China (19°30′N, 110°15′E). Samples were taken from the mangrove forest dominated by *Avicennia marina* (BM1), *Aegiceras corniculatum* (BM2), *Bruguiera sexangula* (BM3), *Xylocarpus mekongensis* (BM4) and *Pongamia pinnata* (BM5), respectively. BM1 and BM2 sited in the seaward edge of the magrove wetland, BM3 and BM4 sited in the inner and BM5 sited in the landedge. Bulk soils, each in triplicate in each location, were collected in December 2011. The three replicates in one location were taken approximately 5 m apart, and then pooled together.

### DNA extraction and 454 pyrosequencing

Microbial DNA was extracted from five soil samples using the FastPrep® SPIN Kit for Soil (MP Biomedicals, U.S.) according to manufacturer’s protocols. The bacterial 16S ribosomal RNA gene were amplified by polymerase chain reaction (95 °C for 2 min, followed by 25 cycles at 95 °C for 30 s, 55 °C for 30 s, and 72 °C for 30 s and a final extension at 72 °C for 5 min) using primers 341F (5′-CCTACGGGAGGCAGCAG-3′)-1073R (5′-ACGAGCTGACGACARCCATG-3′), and the archaeal 16S ribosomal RNA gene were amplified by polymerase chain reaction (95 °C for 2 min, followed by 30 cycles at 95 °C for 30 s, 55 °C for 30 s, and 72 °C for 30 s and a final extension at 72 °C for 5 min) using primers 344F (5′-ACGGGGYGCAGCAGGCGCGA-3′)-915R (5′-GTGCTCCCCCAATTCCT-3′). PCR reactions were performed in a 20 μL mixture containing 4 μL of 5 × FastPfu Buffer, 2 μL of 2.5 mM dNTPs, 0.8 μL of each primer (5 μM), 0.4 μL of FastPfu Polymerase, and 10 ng of template DNA. After purification using the AxyPrep DNA Gel Extraction Kit (Axygen Biosciences, Union City, CA, U.S.) and quantification using QuantiFluor™ -ST (Promega, U.S.), a mixture of amplicons was used for pyrosequencing on a Roche 454 GS FLX+ Titanium platform (Roche 454 Life Sciences, Branford, CT, U.S.) according to standard protocols. The raw reads were deposited into the NCBI Sequence Read Archive (SRA) database (Accession Number: SRP040784 for bacteria and SRP041275 for archaea).

### Processing of pyrosequencing data

The resulting sequences were processed using QIIME (version 1.17)^67^. After removing sequences with average quality score <25 over a 50 bp sliding window and sequences shorter than 200 bp, with homopolymers longer than six nucleotides, and containing ambiguous base calls or incorrect primer sequences, high-quality sequences were produced. Operational Taxonomic Units (OTUs) were clustered with 97% similarity cutoff using UPARSE (version 7.1 http://drive5.com/uparse/)^19,68^ and chimeric sequences were identified and removed using UCHIME. The phylogenetic affiliation of each 16S rRNA gene sequence was analyzed by RDP Classifier (http://rdp.cme.msu.edu/) against the silva (SSU115)16S rRNA database using confidence threshold of 70%^19,32,68^. The beta diversity analysis was carried out using UniFrac to compare the results of PCA at the OTU level with the community ecology package, R-forge (vegan 2.0 package was used to generate a PCA figure)^35,69^.

### Phylogenetic investigation of communities by reconstruction of unobserved states (PICRUSt) analysis

PICRUSt analysis was performed to predict metagenomic functional content based on the software package (PICRUSt v1. 0. 0)^70^. This approach exploits the relationship between phylogeny and function by combining 16 s data with a database of reference genomes (Greengenes) to predict the presence of gene families. The 16S rDNA sequences were clustered into a collection of OTUs using a closed-reference OUT picking protocol (QIIME1.8.0)^71^. The obtained OUT table was normalized by 16S rRNA gene copy number, and then used to predict metagenomic functional content based on the PICRUSt software package^70^. Functional predictions were exported as KEGG orthologs.

## Supplementary information

Daeaset 1

## Data Availability

The datasets generated during the current study are available in the NCBI Sequence Read Archive (SRA) database (Accession Number: SRP040784 for bacteria and SRP041275 for archaea).
