# Supplementary material for: Microbial community structure of soils in Bamenwan mangrove wetland
Source: Sci Rep. 2019 Jun 10;9:8406. doi: 10.1038/s41598-019-44788-x (PMC6557889; doi:10.1038/s41598-019-44788-x)
Supplement: Supplementary file 1 — Daeaset 1 [file 41598_2019_44788_MOESM1_ESM.doc]

**Supplementary material**

Microbial community structure of soils in Bamenwan mangrove wetland

Min Liu^1,2^, Huiqin Huang^2^, Shixiang Bao^2^, Yuhe Tong^1*^

^1^ Hainan Tropical Ocean University, Sanya, China

^2^ Institute of Tropical Biosciences and Biotechnology, Chinese Academy of Tropical Agricultural Sciences, Haikou, China,

**Table S1** Bacterial sequences assignment results on phylum level (%)

|  | BM1 | BM2 | BM3 | BM4 | BM5 |
| --- | --- | --- | --- | --- | --- |
| Acidobacteria | 4.25 | 5.88 | 7.30 | 8.43 | 10.37 |
| Actinobacteria | 10.10 | 8.86 | 24.82 | 16.70 | 48.70 |
| BD1-5 | 0.08 | 0.15 | 0.04 | 0.00 | 0.00 |
| Bacteria_unclassified | 0.11 | 0.41 | 0.34 | 0.30 | 0.05 |
| Bacteroidetes | 6.28 | 1.97 | 1.61 | 2.61 | 0.40 |
| Caldiserica | 0.00 | 0.02 | 0.00 | 0.00 | 0.00 |
| Candidate_division_KB1 | 0.00 | 0.04 | 0.00 | 0.00 | 0.00 |
| Candidate_division_OD1 | 0.00 | 0.11 | 0.00 | 0.00 | 0.17 |
| Candidate_division_OP11 | 0.00 | 0.07 | 0.00 | 0.00 | 0.00 |
| Candidate_division_OP8 | 0.84 | 1.09 | 0.00 | 0.00 | 0.00 |
| Candidate_division_TM7 | 0.00 | 0.05 | 0.03 | 0.08 | 0.29 |
| Candidate_division_WS3 | 0.64 | 1.03 | 0.49 | 0.97 | 0.01 |
| Candidate_division_WS6 | 0.00 | 0.00 | 0.05 | 0.02 | 0.00 |
| Chlamydiae | 0.00 | 0.00 | 0.05 | 0.03 | 0.00 |
| Chlorobi | 0.86 | 1.46 | 1.73 | 2.48 | 0.41 |
| Chloroflexi | 16.30 | 15.28 | 6.54 | 6.57 | 2.99 |
| Cyanobacteria | 3.55 | 4.78 | 0.54 | 0.39 | 0.12 |
| Deferribacteres | 1.31 | 3.39 | 0.19 | 0.49 | 0.00 |
| Deinococcus-Thermus | 0.00 | 0.00 | 0.04 | 0.06 | 0.00 |
| Fibrobacteres | 0.03 | 0.00 | 0.00 | 0.00 | 0.00 |
| Firmicutes | 5.43 | 3.31 | 0.76 | 1.06 | 3.93 |
| Fusobacteria | 0.18 | 0.02 | 0.21 | 0.16 | 0.00 |
| Gemmatimonadetes | 1.50 | 1.18 | 2.98 | 4.07 | 0.27 |
| JL-ETNP-Z39 | 0.04 | 0.16 | 0.00 | 0.00 | 0.00 |
| Nitrospirae | 2.06 | 4.35 | 0.83 | 1.55 | 0.02 |
| Planctomycetes | 0.10 | 0.15 | 0.00 | 0.00 | 0.04 |
| Proteobacteria | 44.20 | 43.27 | 51.40 | 53.53 | 31.53 |
| SHA-109 | 0.06 | 0.01 | 0.00 | 0.00 | 0.02 |
| Spirochaetae | 1.56 | 2.68 | 0.01 | 0.27 | 0.22 |
| TA06 | 0.39 | 0.22 | 0.00 | 0.01 | 0.00 |
| TM6 | 0.09 | 0.05 | 0.04 | 0.04 | 0.02 |
| Tenericutes | 0.06 | 0.00 | 0.00 | 0.03 | 0.00 |
| Verrucomicrobia | 0.00 | 0.00 | 0.03 | 0.14 | 0.09 |
| WD272 | 0.00 | 0.00 | 0.00 | 0.01 | 0.32 |

**Table S2** Bacterial sequences assignment results on class level (%)

|  | BM1 | BM2 | BM3 | BM4 | BM5 |
| --- | --- | --- | --- | --- | --- |
| Acidobacteria | 4.25 | 5.88 | 7.30 | 8.43 | 10.37 |
| Actinobacteria | 10.10 | 8.86 | 24.82 | 16.70 | 48.70 |
| Alphaproteobacteria | 6.99 | 4.91 | 27.14 | 23.87 | 24.59 |
| Anaerolineae | 5.94 | 7.04 | 0.68 | 1.05 | 0.04 |
| Ardenticatenia | 0.42 | 1.05 | 0.10 | 0.02 | 0.00 |
| BD1-5 | 0.08 | 0.15 | 0.04 | 0.00 | 0.00 |
| BD2-2 | 0.52 | 0.45 | 0.08 | 0.01 | 0.00 |
| Bacilli | 1.67 | 0.67 | 0.24 | 0.38 | 3.79 |
| Bacteria_unclassified | 0.11 | 0.41 | 0.34 | 0.30 | 0.05 |
| Bacteroidetes_bacteroidetes_incertae_sedis | 0.25 | 0.00 | 0.00 | 0.00 | 0.00 |
| Bacteroidetes_unclassified | 0.00 | 0.00 | 0.03 | 0.13 | 0.00 |
| Bacteroidia | 0.03 | 0.07 | 0.04 | 0.00 | 0.00 |
| Betaproteobacteria | 0.11 | 0.45 | 1.66 | 1.62 | 0.67 |
| Caldilineae | 0.38 | 0.33 | 0.00 | 0.01 | 0.00 |
| Caldisericia | 0.00 | 0.02 | 0.00 | 0.00 | 0.00 |
| Candidate_division_KB1 | 0.00 | 0.04 | 0.00 | 0.00 | 0.00 |
| Candidate_division_OD1 | 0.00 | 0.11 | 0.00 | 0.00 | 0.17 |
| Candidate_division_OP11 | 0.00 | 0.07 | 0.00 | 0.00 | 0.00 |
| Candidate_division_OP8 | 0.84 | 1.09 | 0.00 | 0.00 | 0.00 |
| Candidate_division_TM7 | 0.00 | 0.05 | 0.03 | 0.08 | 0.29 |
| Candidate_division_WS3 | 0.64 | 1.03 | 0.49 | 0.97 | 0.01 |
| Candidate_division_WS6 | 0.00 | 0.00 | 0.05 | 0.02 | 0.00 |
| Chlamydiae | 0.00 | 0.00 | 0.05 | 0.03 | 0.00 |
| Chlorobia | 0.00 | 0.02 | 0.00 | 0.00 | 0.00 |
| Chloroflexi_unclassified | 4.18 | 3.41 | 0.01 | 0.19 | 0.00 |
| Chloroflexi_uncultured | 1.42 | 0.94 | 0.03 | 0.20 | 0.00 |
| Chloroflexia | 0.00 | 0.00 | 0.00 | 0.00 | 0.02 |
| Clostridia | 3.71 | 2.64 | 0.45 | 0.62 | 0.14 |
| Cyanobacteria | 3.55 | 4.78 | 0.54 | 0.39 | 0.12 |
| Cytophagia | 1.50 | 0.27 | 0.75 | 0.92 | 0.00 |
| Deferribacteres | 1.31 | 3.39 | 0.19 | 0.49 | 0.00 |
| Dehalococcoidia | 2.60 | 1.99 | 0.26 | 0.86 | 0.00 |
| Deinococci | 0.00 | 0.00 | 0.04 | 0.06 | 0.00 |
| Deltaproteobacteria | 20.06 | 27.12 | 11.33 | 16.63 | 1.03 |
| Epsilonproteobacteria | 0.28 | 0.02 | 0.09 | 0.42 | 0.00 |
| Erysipelotrichia | 0.05 | 0.00 | 0.06 | 0.06 | 0.00 |
| Fibrobacteria | 0.03 | 0.00 | 0.00 | 0.00 | 0.00 |
| Flavobacteriia | 2.75 | 0.25 | 0.40 | 0.50 | 0.01 |
| Fusobacteriia | 0.18 | 0.02 | 0.21 | 0.16 | 0.00 |
| Gammaproteobacteria | 16.02 | 9.68 | 10.56 | 10.32 | 5.24 |
| Gemmatimonadetes | 1.50 | 1.18 | 2.98 | 4.07 | 0.27 |
| Gitt-GS-136 | 0.00 | 0.00 | 1.28 | 1.37 | 0.10 |
| Ignavibacteria | 0.86 | 1.44 | 1.73 | 2.48 | 0.41 |
| JG30-KF-CM66 | 0.00 | 0.00 | 0.62 | 0.32 | 0.06 |
| JL-ETNP-Z39 | 0.04 | 0.16 | 0.00 | 0.00 | 0.00 |
| KD4-96 | 1.04 | 0.48 | 1.53 | 1.78 | 0.52 |
| Ktedonobacteria | 0.00 | 0.00 | 0.00 | 0.00 | 0.66 |
| MSB-5B2 | 0.23 | 0.00 | 0.00 | 0.00 | 0.00 |
| Milano-WF1B-44 | 0.16 | 0.01 | 0.00 | 0.00 | 0.00 |
| Mollicutes | 0.06 | 0.00 | 0.00 | 0.03 | 0.00 |
| Nitrospira | 2.06 | 4.35 | 0.83 | 1.55 | 0.02 |
| OM190 | 0.05 | 0.11 | 0.00 | 0.00 | 0.00 |
| OPB35_soil_group | 0.00 | 0.00 | 0.00 | 0.04 | 0.00 |
| Phycisphaerae | 0.00 | 0.04 | 0.00 | 0.00 | 0.00 |
| Planctomycetacia | 0.00 | 0.00 | 0.00 | 0.00 | 0.04 |
| Proteobacteria_proteobacteria_incertae_sedis | 0.33 | 0.19 | 0.03 | 0.03 | 0.00 |
| Proteobacteria_unclassified | 0.18 | 0.84 | 0.58 | 0.53 | 0.00 |
| S-BQ2-57_soil_group | 0.00 | 0.00 | 0.00 | 0.09 | 0.00 |
| S085 | 0.06 | 0.00 | 1.29 | 0.40 | 0.02 |
| SB-1 | 0.08 | 0.00 | 0.08 | 0.31 | 0.00 |
| SB-5 | 0.43 | 0.73 | 0.05 | 0.23 | 0.00 |
| SHA-109 | 0.06 | 0.01 | 0.00 | 0.00 | 0.02 |
| SHA-26 | 0.03 | 0.01 | 0.05 | 0.12 | 0.52 |
| Spartobacteria | 0.00 | 0.00 | 0.03 | 0.01 | 0.09 |
| Sphingobacteriia | 0.39 | 0.17 | 0.13 | 0.17 | 0.39 |
| Spirochaetes | 1.56 | 2.68 | 0.01 | 0.27 | 0.22 |
| TA06 | 0.39 | 0.22 | 0.00 | 0.01 | 0.00 |
| TA18 | 0.00 | 0.02 | 0.00 | 0.00 | 0.00 |
| TK10 | 0.00 | 0.00 | 0.00 | 0.00 | 0.06 |
| TM6 | 0.09 | 0.05 | 0.04 | 0.04 | 0.02 |
| Thermomicrobia | 0.01 | 0.01 | 0.68 | 0.26 | 0.97 |
| VC2.1_Bac22 | 0.33 | 0.01 | 0.00 | 0.00 | 0.00 |
| WD272 | 0.00 | 0.00 | 0.00 | 0.01 | 0.32 |
| Zetaproteobacteria | 0.00 | 0.00 | 0.01 | 0.10 | 0.00 |
| pItb-vmat-80 | 0.06 | 0.00 | 0.00 | 0.00 | 0.00 |
| vadinHA17 | 0.01 | 0.01 | 0.06 | 0.33 | 0.00 |
| vadinHA49 | 0.05 | 0.00 | 0.00 | 0.00 | 0.00 |

**Table S3** Bacterial sequences assignment results on order level (%)

|  | BM1 | BM2 | BM3 | BM4 | BM5 |
| --- | --- | --- | --- | --- | --- |
| 34P16 | 0.00 | 0.00 | 0.03 | 0.01 | 0.00 |
| 43F-1404R | 0.08 | 0.25 | 0.93 | 1.23 | 0.00 |
| AKYG1722 | 0.00 | 0.01 | 0.48 | 0.04 | 0.04 |
| ARKDMS-49 | 0.00 | 0.04 | 0.00 | 0.00 | 0.00 |
| AT-s3-28 | 0.08 | 0.04 | 0.01 | 0.09 | 0.00 |
| Acidimicrobiales | 4.50 | 2.11 | 7.77 | 4.10 | 3.10 |
| Acidobacteriaholophagaeincertaesedis | 0.00 | 0.04 | 0.00 | 0.01 | 0.00 |
| Acidobacteria | 0.49 | 0.62 | 0.66 | 0.47 | 0.00 |
| Acidobacteriales | 0.00 | 0.00 | 0.28 | 0.32 | 1.43 |
| Actinobacteria | 1.79 | 0.76 | 1.46 | 0.73 | 0.30 |
| Alphaproteobacteria_unclassified | 0.01 | 0.00 | 0.34 | 0.24 | 0.00 |
| Alteromonadales | 1.01 | 0.21 | 0.52 | 0.21 | 0.00 |
| Anaerolineales | 5.94 | 7.04 | 0.68 | 1.05 | 0.04 |
| Ardenticatenia uncultured | 0.42 | 1.05 | 0.10 | 0.02 | 0.00 |
| BD1-5 | 0.08 | 0.15 | 0.04 | 0.00 | 0.00 |
| BD2-11 terrestrial group | 0.73 | 0.64 | 1.56 | 1.62 | 0.00 |
| BD2-2 | 0.52 | 0.45 | 0.08 | 0.01 | 0.00 |
| Bacillales | 1.67 | 0.67 | 0.24 | 0.38 | 3.79 |
| Bacteria_unclassified | 0.11 | 0.41 | 0.34 | 0.30 | 0.05 |
| Bacteroidales | 0.03 | 0.07 | 0.04 | 0.00 | 0.00 |
| Bacteroidetes_unclassified | 0.00 | 0.00 | 0.03 | 0.13 | 0.00 |
| Bdellovibrionales | 0.34 | 0.10 | 0.18 | 0.16 | 0.00 |
| Betaproteobacteria_unclassified | 0.01 | 0.01 | 0.24 | 0.13 | 0.00 |
| Burkholderiales | 0.05 | 0.11 | 0.00 | 0.00 | 0.09 |
| C0119 | 0.00 | 0.00 | 0.00 | 0.00 | 0.10 |
| CK-1C4-49 | 0.09 | 0.00 | 0.00 | 0.00 | 0.00 |
| Caldilineales | 0.38 | 0.33 | 0.00 | 0.01 | 0.00 |
| Caldisericales | 0.00 | 0.02 | 0.00 | 0.00 | 0.00 |
| Campylobacterales | 0.28 | 0.02 | 0.09 | 0.42 | 0.00 |
| Candidate_division_KB1 | 0.00 | 0.04 | 0.00 | 0.00 | 0.00 |
| Candidate_division_OD1 | 0.00 | 0.11 | 0.00 | 0.00 | 0.17 |
| Candidate_division_OP11 | 0.00 | 0.07 | 0.00 | 0.00 | 0.00 |
| Candidate_division_OP8 | 0.84 | 1.09 | 0.00 | 0.00 | 0.00 |
| Candidate_division_TM7 | 0.00 | 0.05 | 0.03 | 0.08 | 0.29 |
| Candidate_division_WS3 | 0.64 | 1.03 | 0.49 | 0.97 | 0.01 |
| Candidate_division_WS6 | 0.00 | 0.00 | 0.05 | 0.02 | 0.00 |
| Caulobacterales | 0.00 | 0.04 | 0.43 | 0.29 | 0.44 |
| Chlamydiales | 0.00 | 0.00 | 0.05 | 0.03 | 0.00 |
| Chlorobiales | 0.00 | 0.02 | 0.00 | 0.00 | 0.00 |
| Chloroflexales | 0.00 | 0.00 | 0.00 | 0.00 | 0.02 |
| Chloroflexi | 5.60 | 4.35 | 0.04 | 0.39 | 0.00 |
| Chromatiales | 3.46 | 2.78 | 2.66 | 2.68 | 0.00 |
| Chthoniobacterales | 0.00 | 0.00 | 0.03 | 0.01 | 0.09 |
| Clostridiales | 3.71 | 2.55 | 0.45 | 0.62 | 0.14 |
| Coriobacteriales | 0.80 | 0.07 | 0.00 | 0.03 | 0.00 |
| Corynebacteriales | 0.05 | 0.01 | 0.26 | 0.20 | 0.15 |
| Cyanobacteria | 2.47 | 4.06 | 0.44 | 0.22 | 0.12 |
| Cytophagales | 1.47 | 0.24 | 0.58 | 0.78 | 0.00 |
| Deferribacterales | 1.31 | 3.39 | 0.19 | 0.49 | 0.00 |
| Dehalococcoidales | 0.11 | 0.24 | 0.00 | 0.23 | 0.00 |
| Dehalococcoidia_unclassified | 0.19 | 0.19 | 0.05 | 0.08 | 0.00 |
| Deinococcales | 0.00 | 0.00 | 0.04 | 0.06 | 0.00 |
| Deltaproteobacteria_unclassified | 0.01 | 0.45 | 0.04 | 0.06 | 0.00 |
| Desulfarculales | 0.80 | 1.03 | 0.32 | 0.95 | 0.01 |
| Desulfobacterales | 10.51 | 17.08 | 3.13 | 6.29 | 0.02 |
| Desulfuromonadales | 0.67 | 0.14 | 1.29 | 1.57 | 0.00 |
| E01-9C-26_marine_group | 0.01 | 0.04 | 0.00 | 0.00 | 0.00 |
| E6aD10 | 0.00 | 0.00 | 0.04 | 0.00 | 0.00 |
| EC3 | 0.00 | 0.00 | 0.00 | 0.04 | 0.00 |
| Erysipelotrichales | 0.05 | 0.00 | 0.06 | 0.06 | 0.00 |
| Euzebyales | 0.01 | 0.07 | 0.00 | 0.01 | 0.00 |
| FS117-23B-02 | 1.81 | 0.51 | 0.00 | 0.29 | 0.00 |
| FW22 | 0.00 | 0.04 | 0.00 | 0.00 | 0.00 |
| Flavobacteriales | 2.75 | 0.25 | 0.40 | 0.50 | 0.01 |
| Frankiales | 0.04 | 0.09 | 0.57 | 0.59 | 22.20 |
| Fusobacteriales | 0.18 | 0.02 | 0.21 | 0.16 | 0.00 |
| GIF3 | 0.00 | 0.11 | 0.17 | 0.08 | 0.00 |
| GIF9 | 0.25 | 0.38 | 0.00 | 0.04 | 0.00 |
| GR-WP33-30 | 0.10 | 0.27 | 1.52 | 1.01 | 0.04 |
| Gaiellales | 1.08 | 1.61 | 6.41 | 5.94 | 15.31 |
| Gammaproteobacteria_gammaproteobacteria_incertae_sedis | 0.92 | 0.92 | 0.00 | 0.00 | 0.00 |
| Gammaproteobacteria_incertae_sedis | 0.82 | 0.12 | 0.40 | 0.43 | 0.00 |
| Gammaproteobacteria_unclassified | 1.08 | 2.61 | 0.76 | 0.80 | 0.31 |
| Gastranaerophilales | 0.06 | 0.22 | 0.01 | 0.14 | 0.00 |
| Gemmatimonadales | 0.13 | 0.38 | 0.54 | 1.13 | 0.27 |
| Gemmatimonadetes | 0.64 | 0.15 | 0.88 | 1.31 | 0.00 |
| Gitt-GS-136 | 0.00 | 0.00 | 1.28 | 1.37 | 0.10 |
| HOC36 | 0.18 | 0.46 | 0.00 | 0.00 | 0.00 |
| Halanaerobiales | 0.00 | 0.09 | 0.00 | 0.00 | 0.00 |
| Haloplasmatales | 0.06 | 0.00 | 0.00 | 0.03 | 0.00 |
| Hydrogenophilales | 0.00 | 0.12 | 0.04 | 0.00 | 0.00 |
| Ignavibacteriales | 0.86 | 1.44 | 1.73 | 2.48 | 0.41 |
| JG30-KF-AS9 | 0.00 | 0.00 | 0.00 | 0.00 | 0.14 |
| JG30-KF-CM66 | 0.00 | 0.00 | 0.62 | 0.32 | 0.06 |
| JL-ETNP-Z39 | 0.04 | 0.16 | 0.00 | 0.00 | 0.00 |
| KD4-96 | 1.04 | 0.48 | 1.53 | 1.78 | 0.52 |
| KI89A_clade | 0.30 | 0.02 | 0.34 | 0.58 | 0.01 |
| Kineosporiales | 0.01 | 3.49 | 0.06 | 0.08 | 0.06 |
| Ktedonobacterales | 0.00 | 0.00 | 0.00 | 0.00 | 0.42 |
| Legionellales | 0.00 | 0.00 | 0.04 | 0.03 | 0.06 |
| MD2904-B13 | 0.00 | 0.01 | 0.52 | 0.65 | 0.00 |
| MSB-5B2 | 0.23 | 0.00 | 0.00 | 0.00 | 0.00 |
| MSBL5 | 0.00 | 0.19 | 0.01 | 0.08 | 0.00 |
| Mariprofundales | 0.00 | 0.00 | 0.01 | 0.10 | 0.00 |
| Micrococcales | 0.34 | 0.06 | 0.84 | 0.48 | 0.04 |
| Micromonosporales | 0.03 | 0.24 | 0.10 | 0.13 | 0.60 |
| Milano-WF1B-44 | 0.16 | 0.01 | 0.00 | 0.00 | 0.00 |
| Myxococcales | 0.93 | 1.21 | 1.53 | 1.59 | 0.96 |
| NKB5 | 0.09 | 0.04 | 0.06 | 0.03 | 0.00 |
| Nitrosomonadales | 0.01 | 0.17 | 0.92 | 0.95 | 0.32 |
| Nitrospira | 2.06 | 4.35 | 0.83 | 1.55 | 0.02 |
| OCS116_clade | 0.00 | 0.00 | 0.12 | 0.09 | 0.00 |
| OM190 | 0.05 | 0.11 | 0.00 | 0.00 | 0.00 |
| OPB35_soil_group | 0.00 | 0.00 | 0.00 | 0.04 | 0.00 |
| Oceanospirillales | 0.00 | 0.00 | 0.01 | 0.03 | 0.00 |
| Order_II | 0.03 | 0.04 | 0.17 | 0.14 | 0.00 |
| PeM15 | 0.59 | 0.05 | 0.01 | 0.07 | 0.02 |
| Phycisphaerales | 0.00 | 0.04 | 0.00 | 0.00 | 0.00 |
| Planctomycetales | 0.00 | 0.00 | 0.00 | 0.00 | 0.04 |
| Propionibacteriales | 0.82 | 0.09 | 0.17 | 0.29 | 0.95 |
| Proteobacteria_unclassified | 0.18 | 0.84 | 0.58 | 0.53 | 0.00 |
| Pseudomonadales | 0.03 | 0.00 | 0.00 | 0.00 | 0.00 |
| Pseudonocardiales | 0.00 | 0.00 | 0.01 | 0.00 | 0.22 |
| Rhizobiales | 5.82 | 3.90 | 21.62 | 19.54 | 8.66 |
| Rhodobacterales | 0.42 | 0.11 | 0.14 | 0.30 | 0.00 |
| Rhodospirillales | 0.58 | 0.59 | 3.60 | 2.44 | 14.96 |
| Rickettsiales | 0.08 | 0.00 | 0.04 | 0.00 | 0.06 |
| Rubrobacterales | 0.00 | 0.00 | 0.00 | 0.00 | 0.07 |
| Run-SP154 | 0.19 | 0.04 | 0.00 | 0.00 | 0.00 |
| S-BQ2-57_soil_group | 0.00 | 0.00 | 0.00 | 0.09 | 0.00 |
| S085 | 0.06 | 0.00 | 1.29 | 0.40 | 0.02 |
| SAR324_clade(Marine_group_B) | 0.06 | 0.21 | 0.04 | 0.08 | 0.00 |
| SB-1 | 0.08 | 0.00 | 0.08 | 0.31 | 0.00 |
| SB-5 | 0.43 | 0.73 | 0.05 | 0.23 | 0.00 |
| SC-I-84 | 0.00 | 0.00 | 0.10 | 0.07 | 0.15 |
| SHA-109 | 0.06 | 0.01 | 0.00 | 0.00 | 0.02 |
| SHA-26 | 0.03 | 0.01 | 0.05 | 0.12 | 0.52 |
| SZB30 | 0.25 | 0.05 | 0.00 | 0.00 | 0.00 |
| Salinisphaerales | 0.00 | 0.42 | 0.04 | 0.08 | 0.00 |
| Sh765B-AG-111 | 0.01 | 0.10 | 0.00 | 0.00 | 0.00 |
| Sh765B-TzT-29 | 1.06 | 1.47 | 1.65 | 2.08 | 0.00 |
| Solirubrobacterales | 0.60 | 0.32 | 7.14 | 4.06 | 5.25 |
| Sphaerobacterales | 0.01 | 0.00 | 0.21 | 0.21 | 0.83 |
| Sphingobacteriales | 0.39 | 0.17 | 0.13 | 0.17 | 0.39 |
| Sphingomonadales | 0.09 | 0.26 | 0.83 | 0.97 | 0.47 |
| Spirochaetales | 1.56 | 2.68 | 0.01 | 0.27 | 0.22 |
| Streptomycetales | 0.01 | 0.01 | 0.19 | 0.06 | 0.62 |
| Streptosporangiales | 0.00 | 0.00 | 0.05 | 0.00 | 0.00 |
| Subgroup_10 | 0.33 | 0.21 | 0.28 | 0.17 | 0.00 |
| Subgroup_11 | 0.00 | 0.00 | 0.03 | 0.06 | 0.00 |
| Subgroup_13 | 0.05 | 0.02 | 0.90 | 1.41 | 1.51 |
| Subgroup_15 | 0.00 | 0.00 | 0.10 | 0.04 | 0.00 |
| Subgroup_17 | 0.91 | 1.77 | 0.86 | 1.08 | 0.00 |
| Subgroup_18 | 0.54 | 1.03 | 0.30 | 0.66 | 0.00 |
| Subgroup_2 | 0.00 | 0.00 | 0.66 | 1.10 | 5.49 |
| Subgroup_21 | 0.55 | 0.32 | 0.17 | 0.28 | 0.00 |
| Subgroup_23 | 0.94 | 1.34 | 0.04 | 0.17 | 0.00 |
| Subgroup_3 | 0.00 | 0.00 | 0.49 | 0.12 | 1.22 |
| Subgroup_4 | 0.00 | 0.02 | 0.06 | 0.18 | 0.04 |
| Subgroup_5 | 0.00 | 0.00 | 0.17 | 0.18 | 0.07 |
| Subgroup_6 | 0.08 | 0.11 | 1.68 | 1.58 | 0.61 |
| Subgroup_7 | 0.01 | 0.00 | 0.24 | 0.40 | 0.01 |
| Subgroup_9 | 0.28 | 0.32 | 0.36 | 0.12 | 0.00 |
| SubsectionI | 0.83 | 0.37 | 0.09 | 0.02 | 0.00 |
| SubsectionII | 0.08 | 0.07 | 0.00 | 0.00 | 0.00 |
| SubsectionIII | 0.11 | 0.05 | 0.00 | 0.00 | 0.00 |
| Sva0071 | 0.06 | 0.01 | 0.21 | 0.07 | 0.00 |
| Sva0485 | 1.62 | 1.11 | 0.05 | 0.07 | 0.00 |
| Syntrophobacterales | 3.87 | 3.79 | 0.64 | 1.55 | 0.00 |
| TA06 | 0.39 | 0.22 | 0.00 | 0.01 | 0.00 |
| TA18 | 0.00 | 0.02 | 0.00 | 0.00 | 0.00 |
| TK10 | 0.00 | 0.00 | 0.00 | 0.00 | 0.06 |
| TM6 | 0.09 | 0.05 | 0.04 | 0.04 | 0.02 |
| TRA3-20 | 0.04 | 0.02 | 0.36 | 0.48 | 0.11 |
| Thermomicrobia_unclassified | 0.00 | 0.00 | 0.00 | 0.00 | 0.10 |
| Thiotrichales | 0.30 | 0.02 | 0.32 | 0.53 | 0.00 |
| Unknown_Order | 0.58 | 0.19 | 0.03 | 0.03 | 0.00 |
| VC2.1_Bac22 | 0.33 | 0.01 | 0.00 | 0.00 | 0.00 |
| Vibrionales | 0.01 | 0.00 | 0.12 | 0.09 | 0.00 |
| WD272 | 0.00 | 0.00 | 0.00 | 0.01 | 0.32 |
| Xanthomonadales | 7.23 | 1.92 | 4.55 | 4.05 | 4.85 |
| d142 | 0.00 | 0.04 | 0.00 | 0.00 | 0.00 |
| pItb-vmat-80 | 0.06 | 0.00 | 0.00 | 0.00 | 0.00 |
| possible_order_07 | 0.03 | 0.00 | 0.00 | 0.00 | 0.00 |
| vadinBA26 | 0.23 | 0.25 | 0.03 | 0.06 | 0.00 |
| vadinHA17 | 0.01 | 0.01 | 0.06 | 0.33 | 0.00 |
| vadinHA49 | 0.05 | 0.00 | 0.00 | 0.00 | 0.00 |

**Table S4** Archaeal sequences assignment results on order level (%)

| Taxon | BM1 | BM2 | BM3 | BM4 | BM5 |
| --- | --- | --- | --- | --- | --- |
| 20a-9 | 0.20 | 0.60 | 0.00 | 0.00 | 0.00 |
| AK59 | 0.80 | 0.10 | 0.00 | 0.00 | 0.00 |
| AK8 | 0.10 | 0.10 | 0.00 | 0.00 | 0.00 |
| Archaea_unclassified | 1.10 | 0.80 | 0.00 | 0.20 | 0.00 |
| Euryarchaeota_unclassified | 0.80 | 1.30 | 0.00 | 0.10 | 0.00 |
| Group_C3 | 14.80 | 21.10 | 1.40 | 7.20 | 0.00 |
| Halobacteriales | 8.60 | 20.80 | 8.10 | 7.50 | 2.60 |
| Marine_Benthic_Group_A | 0.30 | 0.70 | 0.10 | 0.00 | 0.00 |
| Marine_Benthic_Group_B | 13.60 | 14.20 | 1.00 | 12.60 | 0.00 |
| Marine_Group_I | 0.00 | 0.00 | 1.40 | 1.30 | 0.00 |
| Marine_Group_I_unclassified | 0.00 | 0.00 | 0.60 | 1.00 | 0.00 |
| Methanobacteriales | 0.50 | 0.50 | 0.10 | 0.00 | 0.00 |
| Methanocellales | 0.40 | 0.60 | 0.10 | 0.10 | 0.00 |
| Methanococcales | 0.00 | 0.00 | 0.10 | 0.20 | 0.00 |
| Methanomicrobiales | 0.80 | 0.20 | 0.10 | 0.20 | 0.00 |
| Methanosarcinales | 2.40 | 0.90 | 6.60 | 13.90 | 0.10 |
| Miscellaneous_Crenarchaeotic_Group | 36.20 | 16.80 | 2.40 | 8.40 | 0.00 |
| Soil_Crenarchaeotic_Group(SCG) | 0.90 | 1.30 | 75.60 | 43.00 | 2.40 |
| South_African_Gold_Mine_Gp_1 | 0.00 | 0.00 | 0.20 | 0.20 | 14.80 |
| Terrestrial_Hot_Spring_Gp(THSCG) | 0.60 | 0.20 | 0.00 | 0.00 | 0.00 |
| Terrestrial_Group | 0.00 | 0.00 | 0.50 | 0.30 | 68.70 |
| Thaumarchaeota_unclassified | 0.60 | 1.40 | 0.00 | 0.00 | 3.00 |
| Thermoplasmatales | 17.20 | 18.40 | 0.40 | 2.00 | 8.40 |
| Unknown_Order | 0.00 | 0.10 | 1.20 | 1.70 | 0.00 |

(a)


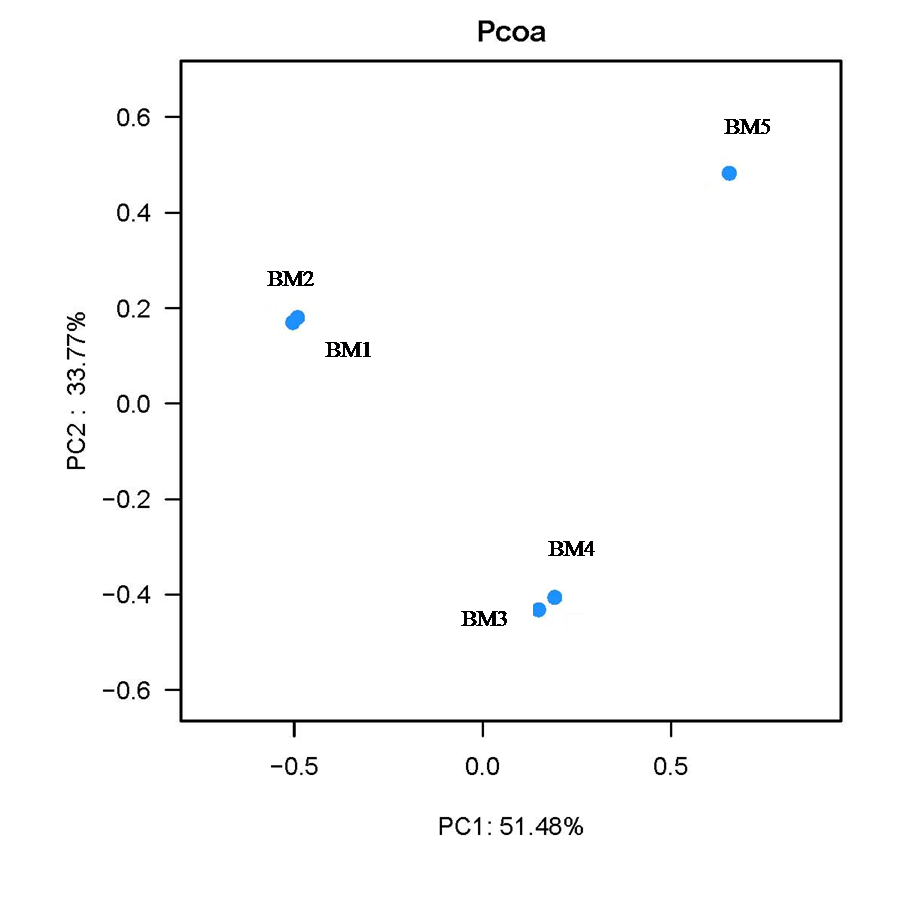


(b)


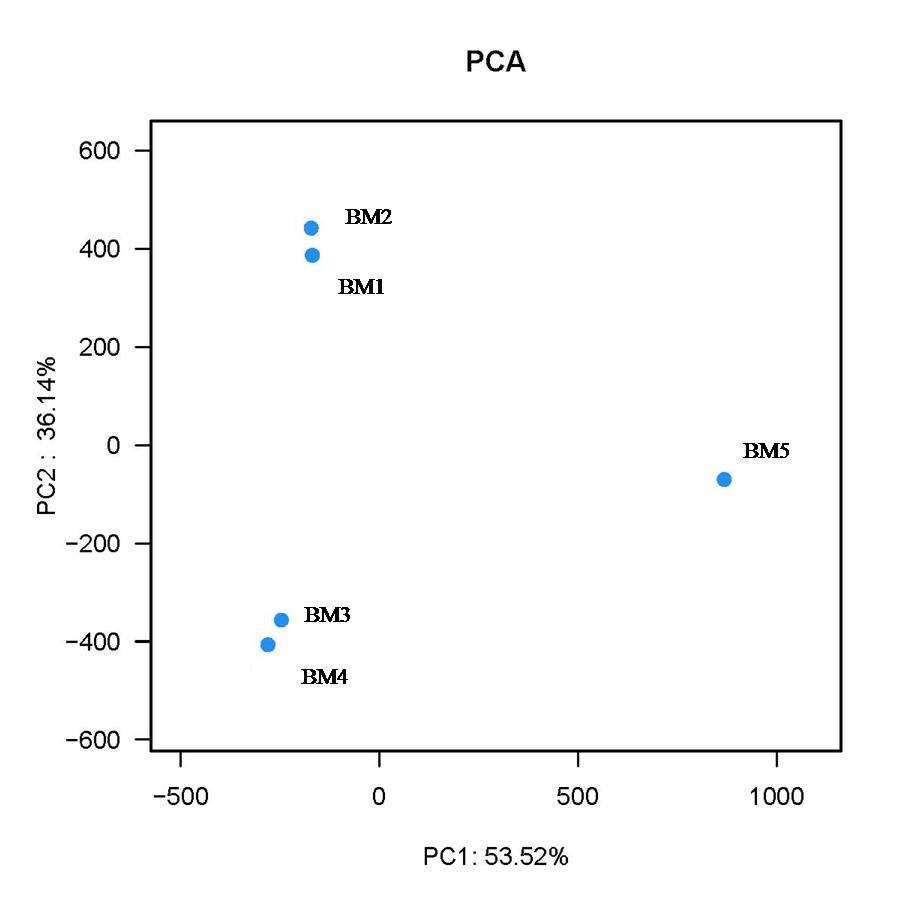


(c)


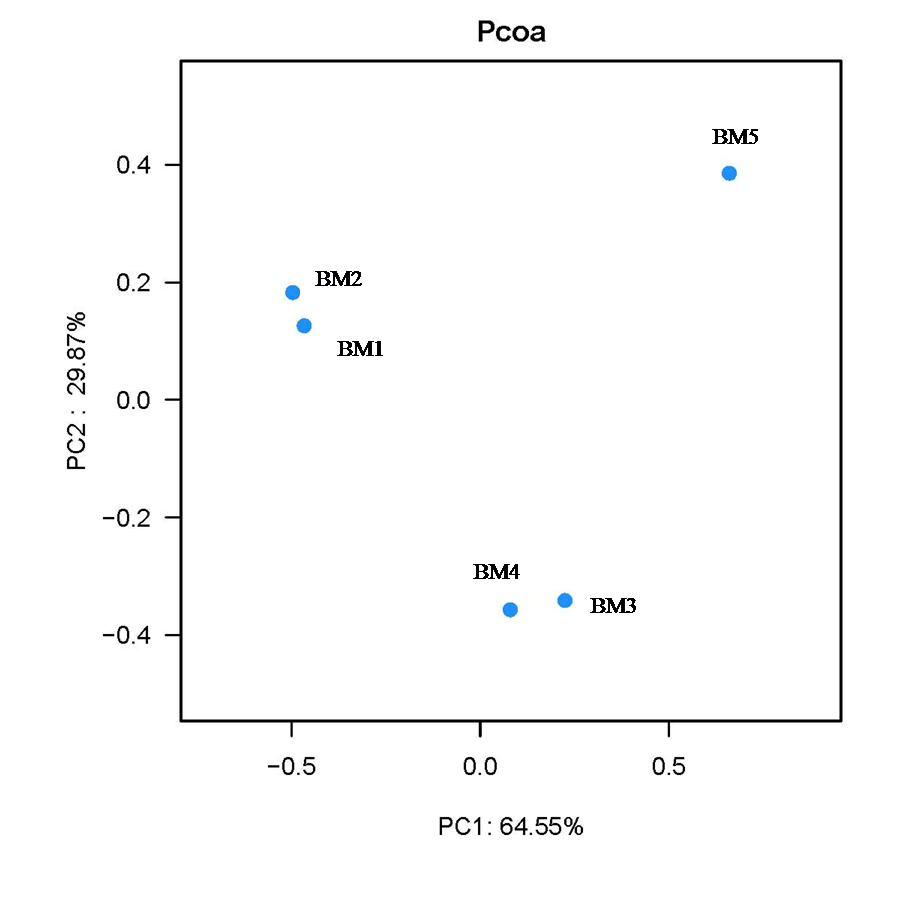


(d)


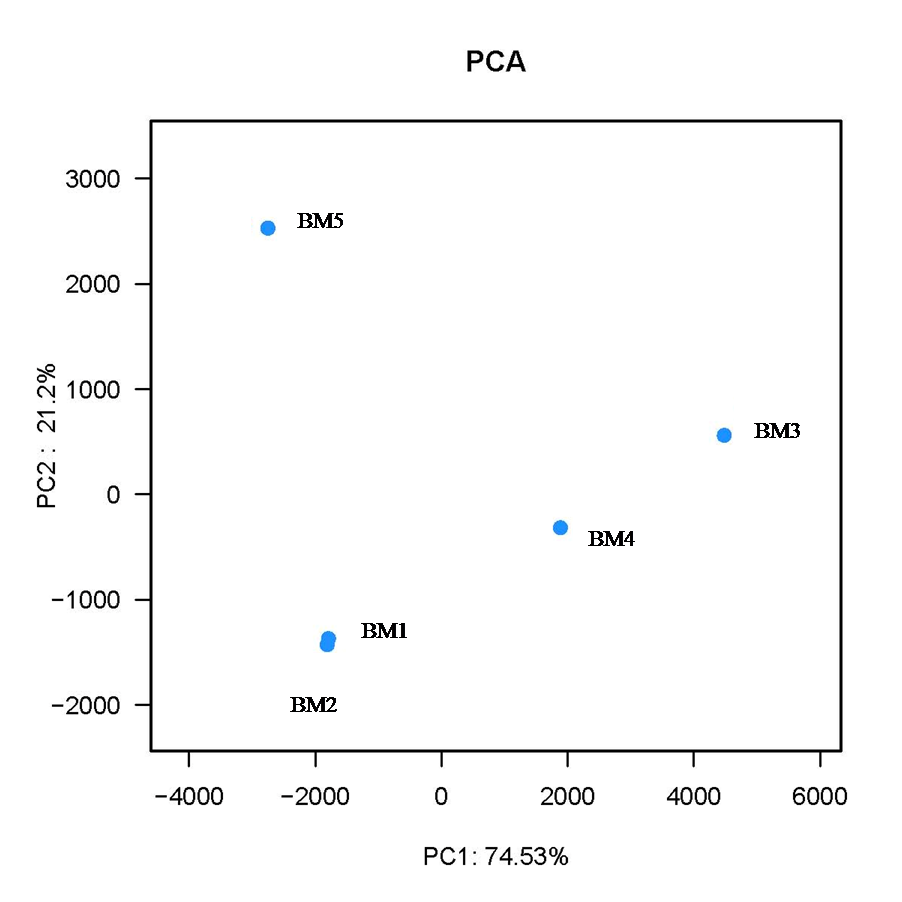


(e)


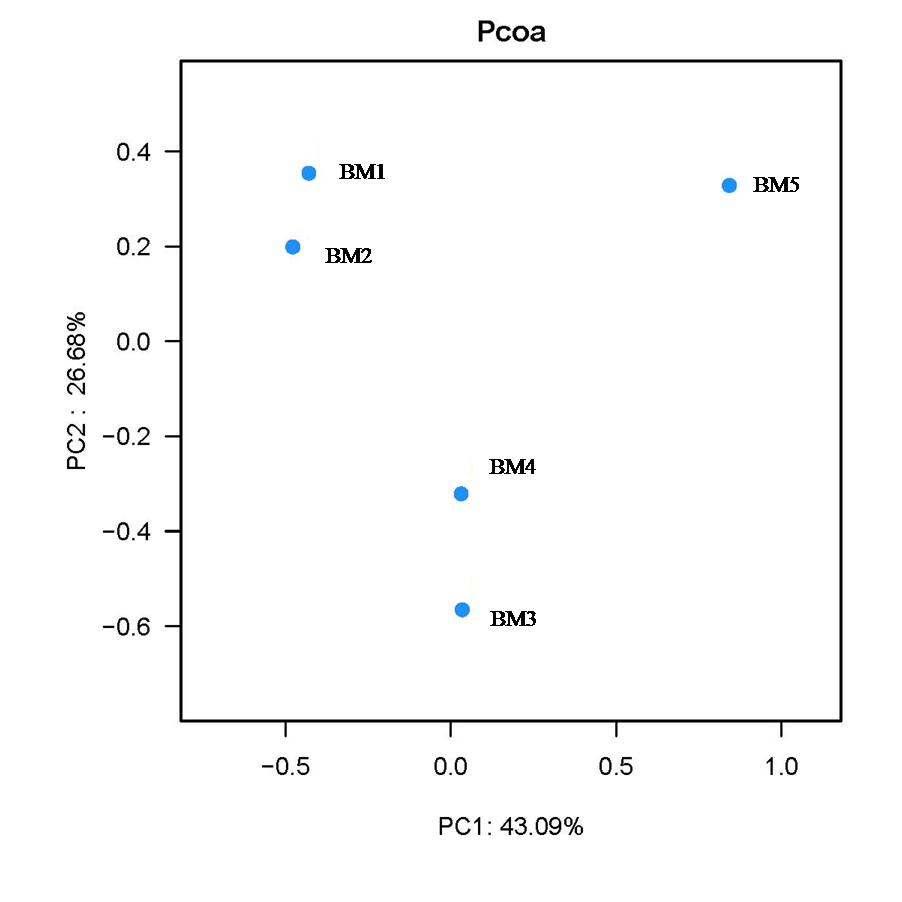


(f)


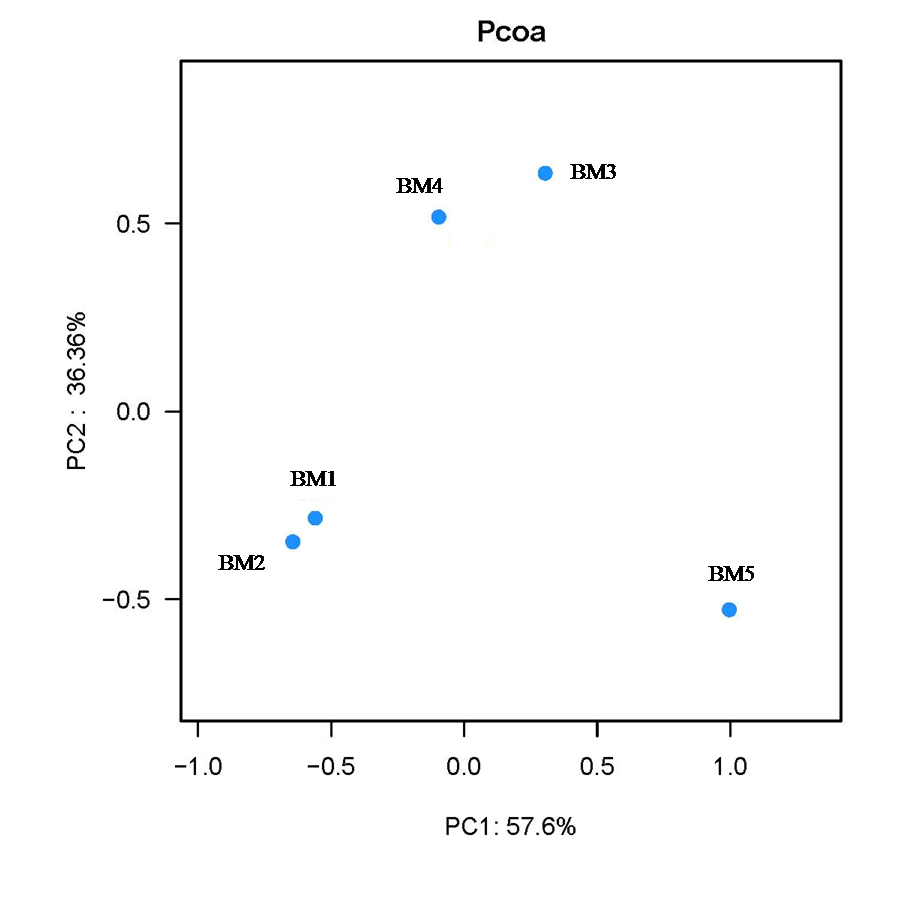


**Fig. S1** Principal component analysis (PCA) at OTUs levels and principal coordinate analysis (PCoA) analysis. (a) PCA result of bacteria, (b) unweighted UniFrac PCoA result of bacteria, (c) weighted UniFrac PCoA result of bacteria, (d) PCA result of archaea, (e) unweighted UniFrac PCoA result of archaea, and (f) weighted UniFrac PCoA result of archaea. PC1 and PC2 were used to plot all PCA and PCoA results.
